# LncRNA LOXL1‐AS1 facilitates the tumorigenesis and stemness of gastric carcinoma via regulation of miR‐708‐5p/USF1 pathway

**DOI:** 10.1111/cpr.12687

**Published:** 2019-08-29

**Authors:** Qi Sun, Jian Li, Fan Li, Huanqin Li, Songhua Bei, Xiaohong Zhang, Li Feng

**Affiliations:** ^1^ Endoscopy Center Minhang Hospital, Fudan University Shanghai China

**Keywords:** CSC, gastric cancer, LOXL1‐AS1, miR‐708‐5p, USF1

## Abstract

**Objectives:**

As one of the most life‐threatening malignancies, gastric cancer is the third contributor of cancer mortalities globally. Increasing studies have proven the regulatory roles of lncRNAs in the development of diverse malignant tumours. But little is known about its function and molecular mechanism in gastric carcinoma.

**Materials and methods:**

RT‐qPCR was performed to measure the expression pattern of LOXL1‐AS1 in gastric cancer. To ascertain its definite role, CCK‐8, EdU, Western blot, transwell and sphere formation assays were adopted. RNA pull‐down, RIP, ChIP and luciferase reporter assays were carried out to investigate the molecular mechanism of LOXL1‐AS1 in gastric carcinoma.

**Results:**

LOXL1‐AS1 was highly expressed in tissues and cells of gastric cancer. The upregulation of LOXL1‐AS1 predicted poor prognosis in gastric carcinoma. Our findings demonstrated that LOXL1‐AS1 accelerated the deterioration of gastric cancer by inducing cell proliferation, migration, EMT and stemness. Moreover, the expression of USF1 in gastric cancer was higher than in normal control and LOXL1‐AS1 negatively modulated USF1. Functionally, LOXL1‐AS1 acted as a ceRNA to upregulate USF1 via sponging miR‐708‐5p. Besides, we confirmed USF1 promoted the transcription of stemness marker SOX2. Rescue experiments testified the stimulative role of LOXL1‐AS1/miR‐708‐5p/USF1 pathway in gastric cancer progression. It was also validated that LOXL1‐AS1 facilitated cell growth of gastric carcinoma in vivo.

**Conclusions:**

Our study unravelled that LOXL1‐AS1/miR‐708‐5p/USF1 pathway contributed to the development of gastric cancer.

## 1 INTRODUCTION

Gastric cancer originating from gastric mucosa is one of the most prevailing life‐threatening malignancies around the world.^1^ As the third contributor of cancer mortalities globally, gastric cancer enforces a significant burden on public health.^2^ There are approximately 1 033 000 newly diagnosed cases and 783 000 patients succumbed to gastric carcinoma worldwide.^3^ Among diverse epidemiologic risk factors, Helicobacter pylori infection is the main inducer of gastric cancer.^4^ Although the rapid progresses have been acquired in the treatment of gastric cancer during recent decades, the prognosis of post‐operative patients is extremely poor and 5‐year survival rate of gastric cancer patients is far <10% resulting from metastasis.^5^ Therefore, it is necessary to comprehend the mechanism governing gastric cancer to identify potent targets for clinical therapy.

Cancer stem cells (CSCs), also well‐acknowledged as tumour‐initiating cells, are a group of tumour cells with self‐renewal and unlimited replication ability so that to trigger the initiation and progression of multiple cancers, including gastric carcinoma.^6^, ^7^ A great deal of evidence has proven that CSCs play vital roles in tumorigenicity, metastasis, treatment tolerance and relapse.^8^, ^9^ For the reason that CSC characteristics are of immense significance in the evolution of gastric cancer, elucidation of the molecular mechanisms contributing to CSC properties is urgently needed.

It has been reported that almost 80% of DNAs can be transcribed into RNAs, but far <2% of RNA molecules are translated into proteins.^10^ These RNAs, the lack of protein‐coding potential, are known as non‐coding RNAs (ncRNA).^11^ Long non‐coding RNAs (lncRNA) are a novel class of ncRNAs and generally longer than 200 nucleotides.^12^ Surging evidence has illuminated that lncRNAs are participated in the development of numerous cancers through epigenetic modification, transcriptional or post‐transcriptional modulation and mRNA processing.^13^ LncRNA LOXL1 antisense RNA 1 (LOXL1‐AS1) located on human chromosome 15q24.1 is consisted of 10 781 nucleotides and five exons. A multitude of investigations have validated the oncogenic role of LOXL1‐AS1 in diverse human cancers. For example, lncRNA LOXL1‐AS1 activates the PI3K/AKT pathway to promote cell proliferation and metastasis of medulloblastoma.^14^ LOXL1‐AS1 indicates poor prognosis and facilitates cell proliferation, migration and invasion of osteosarcoma.^15^ LOXL1‐AS1 accelerates prostate cancer progression via targeting miR‐541‐3p/CCND1 axis.^16^ However, the function and latent mechanism of LOXL1‐AS1 in gastric cancer is largely to be clarified.

Hence, the purpose of this study was to investigate the expression pattern and biological significance of LOXL1‐AS1 in gastric cancer. Our results unveiled that LOXL1‐AS1 acted as an oncogene in gastric cancer. Mechanically, LOXL1‐AS1 exerted its performance through modulation of miR‐708‐5p/USF1, which provided a better understanding of LOXL1‐AS1‐mediated gastric cancer progression.

## 2 MATERIALS AND METHODS

### 2.1 Clinical tissue specimens and cell culture

The tumour tissues and paired adjacent non‐cancer tissues were collected from a total of 84 patients diagnosed with gastric cancer at Minhang Hospital, Fudan University. The protocol of this study was approved by the Ethics Committee of Minhang Hospital, Fudan University, and written informed consent was acquired from all recruited patients. None of the participants received antitumour treatment prior to surgical resection. All samples were promptly frozen with liquid nitrogen after excision and preserved at −80°C until further use. Four human gastric cancer cell lines (MKN‐45, AGS, SGC7901 and MGC‐803) and human normal gastric epithelial cell line GES‐1 were procured from American Type Culture Collection (ATCC). All cells were grown in DMEM (Gibco) complemented with 10% FBS (Gibco) at 37°C in the presence of 5% CO_2_.

### 2.2 Cell transfection

The shRNA vectors against LOXL1‐AS1 (sh‐LOXL1‐AS1#1/2/3) or USF1 (sh‐ USF1#1/2/3) were utilized for knockdown of LOXL1‐AS1 or USF1 with scrambled shRNA (sh‐NC) as negative control. For upregulation of LOXL1‐AS1 or USF1, the full length of LOXL1‐AS1 or USF1 was inserted into pcDNA3.1 vectors (Invitrogen) and the empty plasmids served as negative control. To overexpress or downregulate miR‐708‐5p, the mimic and inhibitor of miR‐708‐5p and negative control (NC mimic and NC inhibitor) were bought from GenePharma. Cell transfection was performed with Lipofectamine 2000 (Invitrogen) according to the product manuals.

### 2.3 Real‐time quantitative PCR (RT‐qPCR)

Total RNA from tissues and cells was isolated by using TRIzol reagent (Life Technologies Corporation) obeying the supplier's instructions. Reverse transcription was conducted with the TaqMan RNA Reverse Transcription kit (Applied Biosystems) and TaqMan MicroRNA Reverse Transcription kit (Applied Biosystems). PCR was implemented on the 7500 Fast RT‐PCR System with the One‐Step SYBR Prime‐Script RT‐PCR Kit (TakaraBio). The sequences of main primers were as follows: LOXL1‐AS1 (forward): 5′‐TTCCCATTTACCTGCCCGAAG‐3′, LOXL1‐AS1 (reverse): 5′‐GTCAGCAAACACATGGCAAC‐3′; miR‐708‐5p (forward): 5′‐GGCGCGCAAGGAGCTTACAATC‐3′, miR‐708‐5p (reverse): 5′‐GTGCAGGGTCCGAGGTAT‐3′; USF1 (forward): 5′‐GCTCTATGGAGAGCACCAAGTC‐3′, USF1 (reverse): 5′‐AGACAAGCGGTGGTTACTCTGC‐3′; SOX2 (forward): 5′‐GGGAAATGGGAGGGGTGCAAAAGA‐3′, SOX2 (reverse): 5′‐TTGCGTGAGTGTGGATGGGATTGG‐3′; β‐actin (forward): 5′‐CACCATTGGCAATGAGCGGTTC‐3′, β‐actin (reverse): 5′‐AGGTCTTTGCGGATGTCCACGT‐3′; U6 (forward): 5′‐GCTTCGGCAGCACATATACTAAAAT‐3′, U6 (reverse): 5′‐CGCTTCACGAATTTGCGTGTCAT‐3′. β‐actin and U6 were utilized as endogenous controls. The relative gene expression was quantified by the 2^−ΔΔCt^ method.

### 2.4 Western blot

Total protein extraction was carried out with a RIPA lysis buffer (Beyotime Biotechnology). The protein concentration was determined by the BCA Protein Assay Kit (Pierce Biotechnology). Equivalent proteins were electrophoresed on 10% SDS‐PAGE gel and subsequently transferred to polyvinylidene fluoride (PVDF) membranes. Thereafter, membranes were blocked in 5% skim milk, followed by incubation with primary antibody at 4°C overnight, probed by appropriate secondary antibody at room temperature for 1 hour and then visualized with chemiluminescence molecular imaging system (Bio‐Rad). The following primary antibodies were applied: anti‐USF1 ((ab125020; Abcam), anti‐E‐cadherin (sc‐8426; Santa Cruz), anti‐vimentin (sc‐6260; Santa Cruz), β‐actin (sc‐7963; Santa Cruz), anti‐Nanog (ab109250; Abcam), anti‐SOX2 (ab137385; Abcam) and anti‐OCT4 (ab184665; Abcam). β‐actin served as the loading control.

### 2.5 Cell proliferation assays

For Cell Counting Kit‐8 (CCK‐8) assay, transfected cells were inoculated at a density of 2 × 10^3^ cells/well into 96‐well plates and cultivated for 0, 24, 48, 72 and 96 hours. After different incubation times, each well was added with 20 μL of CCK‐8 reagent (Beyotime Institute of Biotechnology) and cultured for another 2 hours. Then, the absorbance at 450 nm was recorded with a standard microplate reader (Scientific MultiskanMK3, Thermo Scientific).

For 5‐ethynyl‐2′‐deoxyuridine (EdU) assay, cell proliferative ability was estimated with the EdU proliferation detection kit (RiboBio) in the light of the manufacturer's protocol. Briefly, cells were treated with EdU for 2 hours and then stained by 4′,6‐diamidino‐2‐phenylindole (DAPI) (Thermo Fisher Scientific). The images of EdU‐positive cells were captured under a fluorescence microscope (Olympus).

### 2.6 Cell migration assay

Transwell assay was conducted to determine cell migration using an 8‐μm pore size polycarbonate membrane (Costar). After transfection, cells resuspended in serum‐free medium were plated into the upper chamber. The bottom chamber was filled with DEME containing 10% FBS. At 24 hours post‐incubation at 37°C, migrated cells on the lower surface of the membrane were immobilized in methanol, stained by 0.5% crystal violet and counted in five random fields with a microscope.

### 2.7 Subcellular fractionation

NE‐PER™ Nuclear and Cytoplasmic Extraction Reagents (Thermo Fisher Scientific) and the RNeasy Midi Kit (Qiagen) were applied to detach and harvest cytoplasmic and nuclear fractions according to the vender's instructions. Extracted RNAs were subjected to RT‐qPCR analysis to verify the cellular localization of LOXL1‐AS1 with GAPDH as the cytoplasm control and U6 as the nucleus control.

### 2.8 Sphere formation assay

Transfected MKN‐45 and AGS cells were plated in the six‐well ultra‐low attachment plates (Corning). Cells (2 × 105) were cultured in the serum‐free DMEM medium containing 0.4% BSA (Sigma), 2% B27 (BD Pharmingen), 5 μg/mL insulin (Sigma), 20 ng/mL basic fibroblast growth factor (bFGF, Invitrogen) and 20 ng/mL epidermal growth factor (EGF, Invitrogen). Following incubation for 2 weeks at 37°C, the diameter and quantity of spheres were analysed with a light microscope (Nikon) and NIS‐Element F3.0 program (Nikon).

### 2.9 Immunohistochemistry (IHC)

In short, paraffin‐embedded tissues were cut into 4‐μm‐thick slices, dewaxed and rehydrated with graded ethanol. Subsequently, antigen retrieval was performed by using Target Retrieval Solution (Dako) based on the manufacturer's recommendations. The sections were treated with 0.3% hydrogen peroxide and 10% goat serum, probed with the primary antibodies against Ki‐67, E‐cadherin, vimentin and SOX2 overnight and then incubated with second antibodies. The 3,39‐diaminobenzidine (DAB) substrate kit (Vector Laboratories) was employed to detect the expression of proteins. Hematoxylin QS (Vector Laboratories) was applied to counterstain the slides, and the images of all samples were observed with a microscope (Carl Zeissy).

### 2.10 Luciferase reporter assay

The wild‐type and mutant fragments of LOXL1‐AS1 were subcloned into pGL3 plasmids (Promega) to construct LOXL1‐AS1‐WT and LOXL1‐AS1‐Mut. Likewise, the 3′UTR sequences of USF1 containing predicated or mutated miR‐708‐5p binding sites were used to synthesize USF1‐WT or USF1‐Mut vectors. Cells were co‐transfected with miR‐708‐5p mimic, miR‐708‐5p inhibitor or negative control and corresponding reporter plasmids using Lipofectamine 2000 reagent (Invitrogen). At 48 hours after transfection, luciferase activity was estimated with the dual‐luciferase reporter assay system (Promega).

### 2.11 RNA immunoprecipitation (RIP)

Magna RIPTM RNA kit (Millipore) was utilized to carry out RIP assay in conformity with the instructions of manufacturer. Transfected cells were lysed by RIP lysis buffer, and cell extracts were incubated with magnetic beads coated with Ago2 antibody (Millipore) or negative control IgG (Millipore). Afterwards, the beads were washed and treated with Proteinase K to digest proteins. The immunoprecipitated RNA was collected, purified and determined by RT‐qPCR assay.

### 2.12 RNA pull‐down assay

After washing with ice‐cold PBS, cell lysates were obtained using RIP lysis buffer and then transfected with biotinylated LOXL1‐AS1 probe (Bio‐LOXL1‐AS1) or the negative control probe (Bio‐NC). 48 hours later, the lysates were treated with streptavidin magnetic beads (Invitrogen) for 2 hours following the vender's directions. RT‐qPCR assay was conducted to examine the abundance of miRNAs in precipitated complexes bound to the beads.

### 2.13 Chromatin immunoprecipitation (ChIP)

ChIP assay was implemented with the EZ‐ChIP™ Chromatin immunoprecipitation kit (Millipore) in accordance with the directions of the supplier. In brief, cross‐linked chromatins were immunoprecipitated with USF1 antibody (Millipore) or negative control IgG antibody (Millipore). Finally, the expression of SOX2 promoter in precipitated chromatin DNA was detected by RT‐qPCR.

### 2.14 Xenograft experiment

3 × 10^6^ MKN‐45 cells stably transfected with sh‐NC or sh‐LOXL1‐AS1#1 were subcutaneously inoculated into 5‐week‐old BALB/c nude mice to established animal models. Four weeks after injection, mice were euthanized and then the weight of xenografts was tested. Tumour volume was monitored and measured every 4 days. All procedures of animal experiment were approved by the Animal Care and Use Committee of Minhang Hospital, Fudan University, in line with the institutional guidelines.

### 2.15 Statistical analysis

All statistical analyses were implemented with GraphPad Prism 5.0 software (GraphPad, Inc.). Experimental data were shown as the means ± SD, and all assays were repeated at least three times. Comparison between groups was assessed by one‐way ANOVA or Student's *t* test. Spearman's rank correlation analysis was employed to evaluate the associations between study variables. The Kaplan‐Meier method and log‐rank test were utilized to plot and analyse survival curves. Statistical significance was set at *P* < .05.

## 3 RESULTS

### 3.1 High expression of LOXL1‐AS1 reflected poor prognosis of gastric cancer

**Figure 1:**
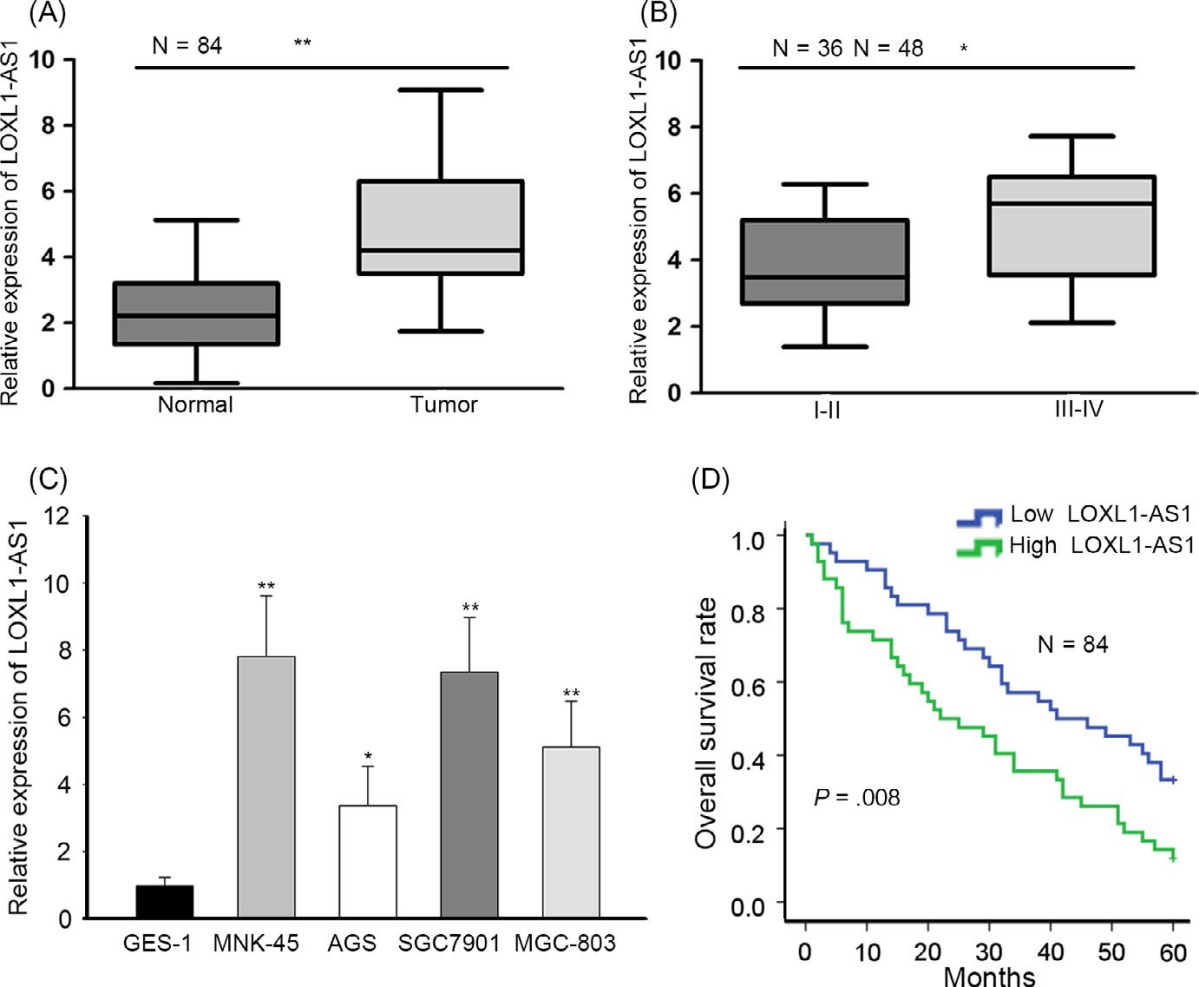
High expression of LOXL1‐AS1 reflected poor prognosis of gastric cancer. A, The expression of LOXL1‐AS1 in gastric cancer tissues (n = 84) and paired non‐cancerous tissues (n = 84) was determined by RT‐qPCR. B, RT‐qPCR analysis of LOXL1‐AS1 level in different TNM stages. C, LOXL1‐AS1 expression in gastric cancer cells (MKN‐45, AGS, SGC7901 and MGC‐803) and normal gastric epithelial cells GES‐1 as detected by RT‐qPCR assay. D, Kaplan‐Meier analysis was used to assess overall survival of gastric cancer patients with high or low LOXL1‐AS1 expression. ^*^ *P* < .05, ^**^ *P* < .01

To disclose the function of LOXL1‐AS1 in gastric cancer, we first conducted RT‐qPCR analysis to investigate its expression pattern in clinical tissues. It was revealed that the level of LOXL1‐AS1 in gastric carcinoma samples was much higher than in matched adjacent tissues (Figure 1A). Furthermore, in contrast with patients at early stages, LOXL1‐AS1 expression was prominently upregulated in patients with advanced gastric cancer (Figure 1B). Consistently, our findings confirmed that LOXL1‐AS1 was highly expressed in gastric cancer cells compared to normal cells (Figure 1C). According to the median of LOXL1‐AS1 expression, the cohort of gastric cancer patients was categorized as high and low expression groups. Kaplan‐Meier analysis exposed the negative association between LOXL1‐AS1 level and the overall survival rate of gastric cancer patients (Figure 1D). Results described indicated that increased LOXL1‐AS1 expression was observed in gastric cancer and closely correlated with poor outcomes of patients with gastric carcinoma.

### 3.2 LOXL1‐AS1 contributed to cell proliferation, migration and EMT in gastric cancer

**Figure 2:**
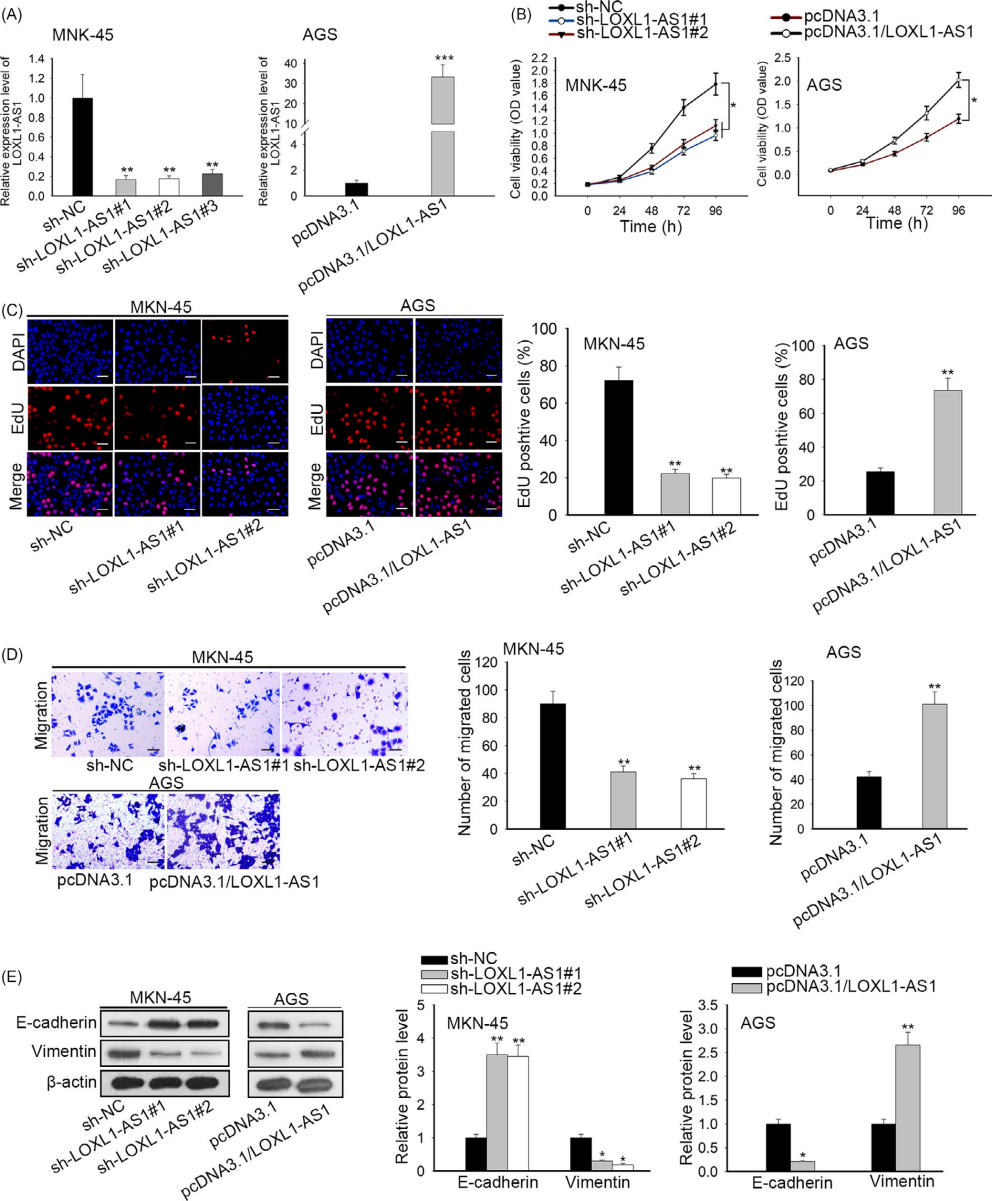
LOXL1‐AS1 contributed to cell proliferation, migration and EMT in gastric cancer. A, The efficiency of transfection in MKN‐45 and AGS cells was determined by RT‐qPCR. B, The CCK‐8 assay was applied to detect cell viability. C, The EdU assay was also conducted to measure cell proliferation in MKN‐45 and AGS cells. D, Cell migratory capacity was tested by transwell assay. E, Western blot analysis was employed to estimate EMT process through examining the expression E‐cadherin and vimentin. ^*^ *P* < .05, ^***^ *P* < .001

Therewith, we made great efforts to illustrate the specific role of LOXL1‐AS1 and implemented functional experiments. Considering that MKN‐45 cells exhibited the highest LOXL1‐AS1 expression and AGS cells presented the lowest level of LOXL1‐AS1, sh‐LOXL1‐AS1#1/2/3 and pcDNA3.1/LOXL1‐AS1, vectors were adopted to overexpress LOXL1‐AS1 in MKN‐45 cells and knock down in AGS cells (Figure 2A). CCK‐8 assay suggested that suppression of LOXL1‐AS1 alleviated cell viability and enhanced expression of LOXL1‐AS1 contributed to the proliferation capacity (Figure 2B). EdU assay validated that the proportion of EdU‐positive cells was dropped by silencing of LOXL1‐AS1 whereas increased by upregulation of LOXL1‐AS1 (Figure 2C). Furthermore, our findings demonstrated that LOXL1‐AS1 knockdown led to the inhibition of cell migration and overexpression of LOXL1‐AS1 displayed the opposite result (Figure 2D). Likewise, the level of E‐cadherin was elevated and vimentin expression was reduced on account of LOXL1‐AS1 depletion, while the enhanced E‐cadherin expression and the lessened level of vimentin were caused by upregulation of LOXL1‐AS1 (Figure 2E). By the large, we concluded that LOXL1‐AS1 induced gastric cancer cell proliferation and metastasis.

### 3.3 LOXL1‐AS1 promoted the maintenance of CSC characteristics in gastric carcinoma

**Figure 3:**
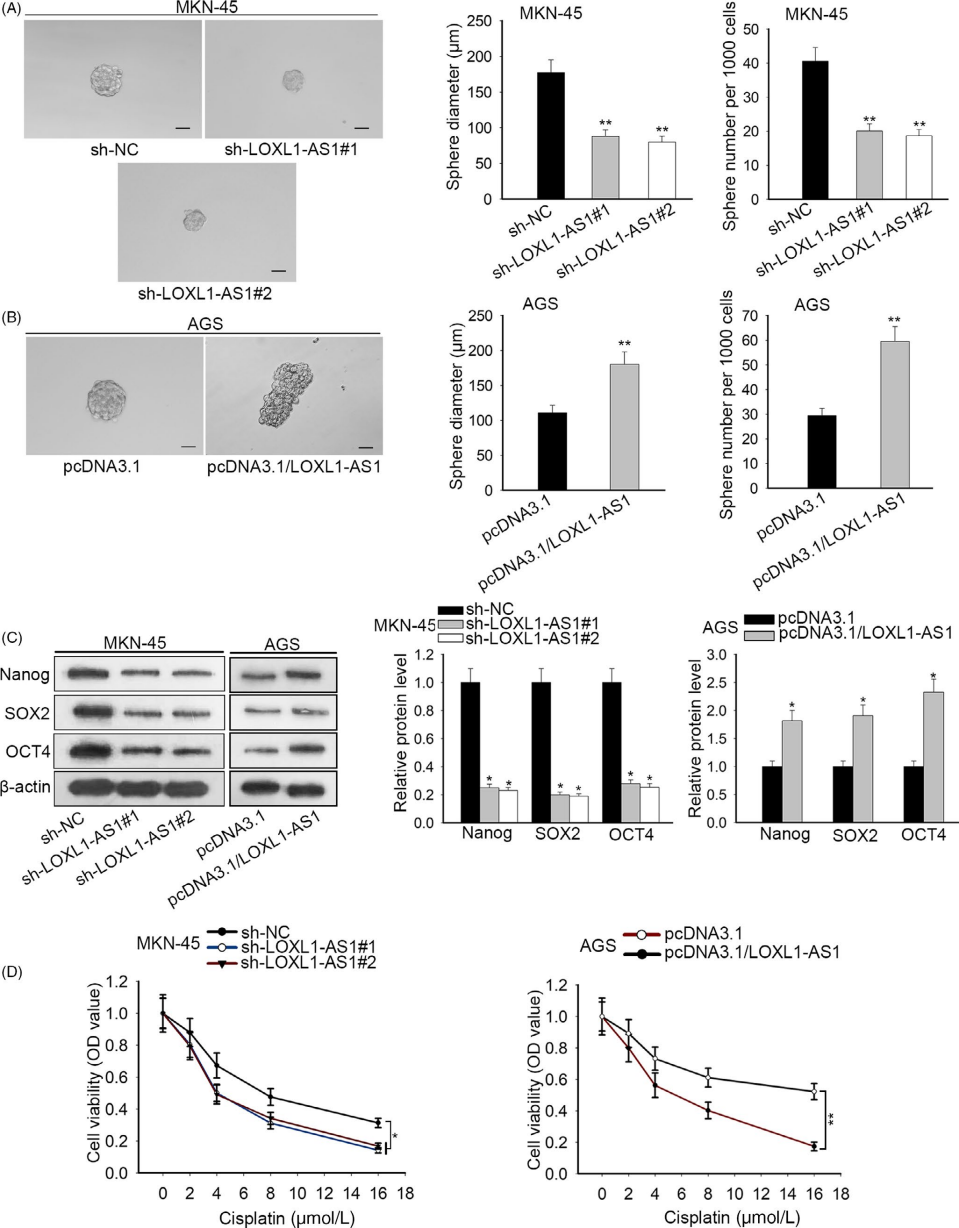
LOXL1‐AS1 promoted the maintenance of CSC characteristics in gastric carcinoma. A, The sphere formation assay was adopted to evaluate the effects of LOXL1‐AS1 depletion on sphere‐forming ability. B, The diameter and quantity of spheres when LOXL1‐AS1 was overexpressed in AGS cells. C, Western blot results of the expression of stem markers (Nanog, SOX2, OCT4) in transfected MKN‐45 and AGS cells. D, CCK8 assay was utilized to assess cell viability after treatment with different doses of cisplatin.^ *^ *P* < .05, ^**^ *P* < .01

Since CSC properties play vital roles in the progression of human malignancy,^17^, ^18^ we estimated the effects of LOXL1‐AS1 on CSC characteristics. Sphere formation assay delineated that inhibition of LOXL1‐AS1 significantly diminished the number and diameter of sphere (Figure 3A). By contrast, ectopic expression of LOXL1‐AS1 contributed to sphere‐forming capability (Figure 3B). Consistently, it was viewed that the expression of stem factors Nanog, SOX2 and OCT4 was weakened by knockdown of LOXL1‐AS1 and enhanced by upregulation of LOXL1‐AS1 (Figure 3C). Given that chemoresistance belongs to the main features of CSC,^19^ the sensitization of transfected MKN‐45 and AGS cells to cisplatin was detected. We observed that MKN‐45 cells with low level of LOXL1‐AS1 were more sensitive to cisplatin treatment, whereas forced expression of LOXL1‐AS1 increased the cisplatin resistance of AGS cells (Figure 3D). Taken together, these data provided strong evidence that LOXL1‐AS1 facilitated the acquisition of CSC characteristics in gastric cancer.

### 3.4 USF1 was the target gene of LOXL1‐AS1/miR‐708‐5p

**Figure 4:**
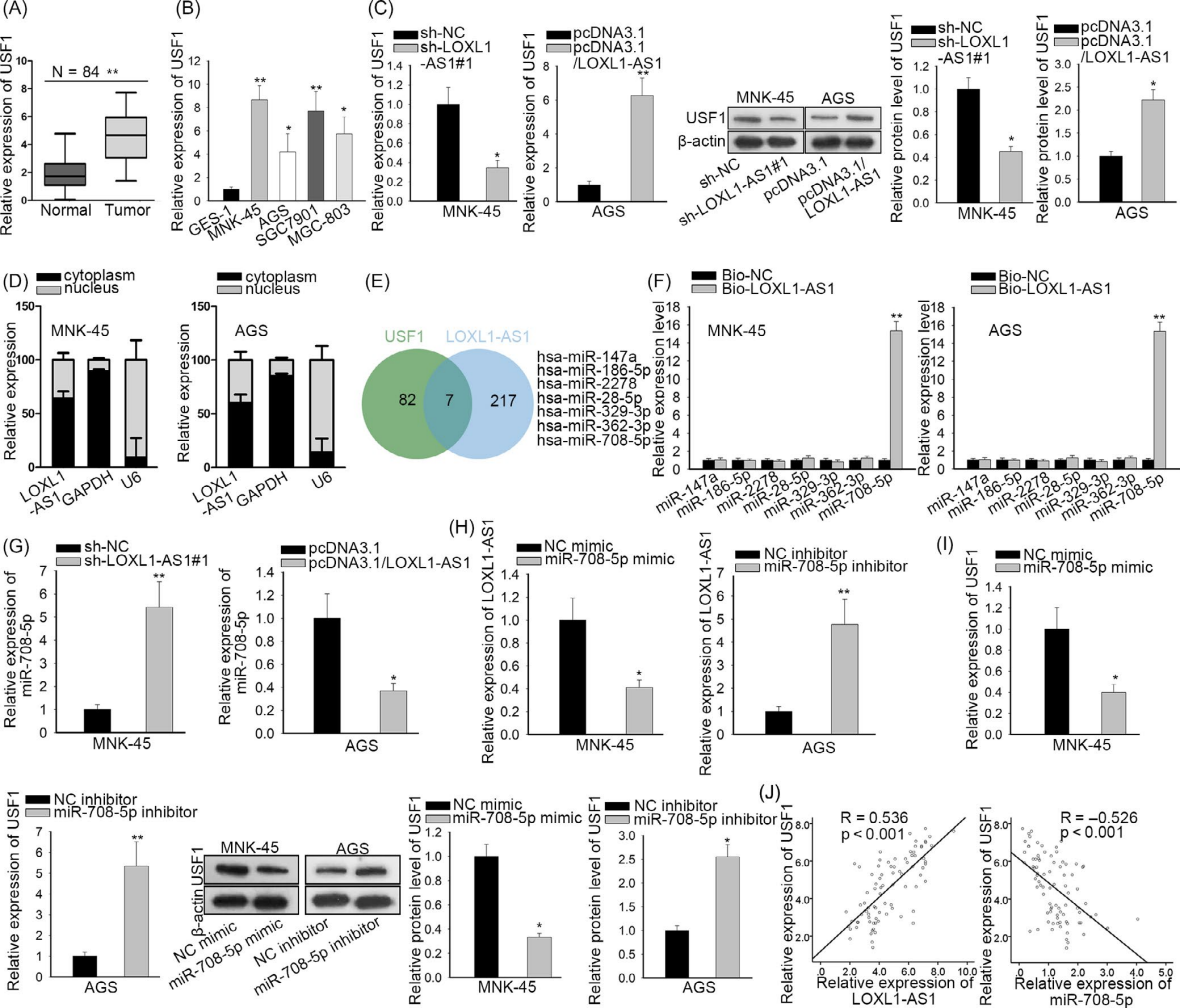
USF1 was the target gene of LOXL1‐AS1/miR‐708‐5p. A‐B, LOXL1‐AS1 expression in gastric carcinoma samples and cells was measured by RT‐qPCR assay. C, After transfection, USF1 expression in MKN‐45 and AGS cells was detected by RT‐qPCR. D, The subcellular position of LOXL1‐AS1 was certified by subcellular fractionation analysis. E, The starBase and DIANA databases were applied to carry out the bioinformatics analysis. F, The relationship between LOXL1‐AS1 and candidate miRNAs was evaluated by RNA pull‐down assay using biotinylated LOXL1‐AS1 probe. G‐H, The RT‐qPCR analysis of miR‐708‐5p and LOXL1‐AS1 in transfected MKN‐45 and AGS cells. I, RT‐qPCR and Western blot were implemented to estimate the impacts of miR‐708‐5p on USF1 expression. J, Spearman's correlation between USF1 and LOXL1‐AS1 or miR‐708‐5p in gastric cancer specimens. ^*^ *P* < .05, ^**^ *P* < .01

In view of the fact that the oncogenic role of USF1 has been reported in diverse cancers,^20^, ^21^, ^22^ we explored the expression of USF1 in gastric cancer tissues and cells. RT‐qPCR analysis showed that USF1 expressed at a higher level in gastric carcinoma samples compared to corresponding normal specimens (Figure 4A). Similarly, the remarkable upregulation of USF1 was observed in gastric cancer cells (Figure 4B). Additionally, it was indicated that depletion of LOXL1‐AS1 attenuated USF1 expression at both mRNA and protein levels, while overexpression of LOXL1‐AS1 heightened the mRNA and protein expression of USF1 (Figure 4C). Subcellular fraction analysis unveiled that LOXL1‐AS1 was preferentially localized in the cytoplasm rather than the nucleus of MKN‐45 and AGS cells (Figure 4D), implying the potential of LOXL1‐AS1 in ceRNA regulatory network.

Hence, bioinformatics analysis was carried out with the assistance of DIANA and starBase databases and we found 7 miRNAs containing the predicted binding sites with both LOXL1‐AS1 and USF1 (Figure 4E). In order to testify the direct binding capability of these miRNAs to LOXL1‐AS1, we performed RNA pull‐down experiments and discovered that only miR‐708‐5p was enriched in complexes pulled down by LOXL1‐AS1 probe compared with other candidate miRNAs (Figure 4F). Besides, miR‐708‐5p expression was notably downregulated in tissues and cells of gastric cancer (Figure S1A,B). As a result, miR‐708‐5p was chosen for the subsequent investigations. RT‐qPCR assay manifested that knockdown of LOXL1‐AS1 caused the increase of miR‐708‐5p expression and upregulation of LOXL1‐AS1 led to the decreased miR‐708‐5p level (Figure 4G). Then, miR‐708‐5p was overexpressed in MKN‐45 cells and silenced in AGS cells using miR‐708‐5p mimic and inhibitor (Figure S1C). Concordantly, suppression of miR‐708‐5p promoted LOXL1‐AS1 expression and miR‐708‐5p upregulation produced the opposite impact (Figure 4H). Furthermore, the mRNA and protein levels of USF1 were boosted by silencing miR‐708‐5p whereas repressed by overexpressing miR‐708‐5p (Figure 4I). Correlation analysis disclosed the positive association between USF1 and LOXL1‐AS1 as well as the negative correlation between USF1 and miR‐708‐5p in clinical tumour tissues (Figure 4J). Collectively, USF1 was regulated by LOXL1‐AS1 and miR‐708‐5p.

### 3.5 LOXL1‐AS1 sponged miR‐708‐5p to upregulate the expression of USF1 in gastric carcinoma

**Figure 5:**
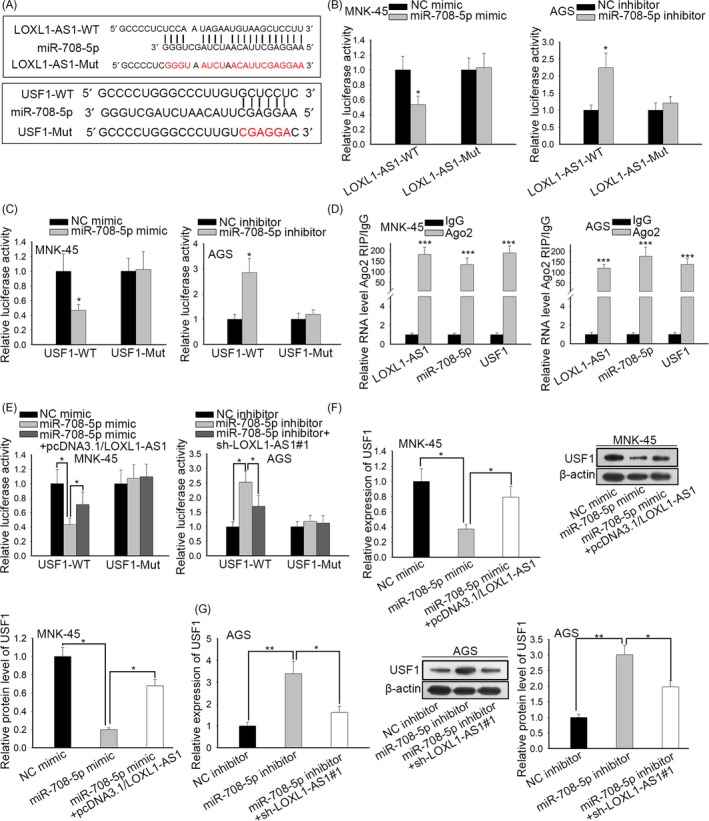
LOXL1‐AS1 sponged miR‐708‐5p to upregulate the expression of USF1 in gastric carcinoma. A, The speculated binding sites of miR‐708‐5p for LOXL1‐AS1 and USF1 by browsing online bioinformatics websites (starBase and DIANA). B‐C, The luciferase reporter assays were performed to measure the luciferase activities of LOXL1‐AS1 and USF1 in response to miR‐708‐5p mimic or miR‐708‐5p inhibitor. D‐E, The interplay between LOXL1‐AS1, miR‐708‐5p and USF1 was further confirmed by RIP and luciferase reporter assays. F‐G, The regulatory influences of LOXL1‐AS1 and miR‐708‐5p on USF1 mRNA and protein levels were verified by RT‐qPCR assay and Western blot. ^*^ *P* < .05, ^***^ *P* < .001

By browsing DIANA and starBase websites, we uncovered the speculated binding sites of miR‐708‐5p for LOXL1‐AS1 and USF1 (Figure 5A). Luciferase assays unravelled that only the luciferase activities of LOXL1‐AS1‐WT and USF1‐WT were impaired by overexpression of miR‐708‐5p and fortified by depletion of miR‐708‐5p, while those of LOXL1‐AS1‐Mut and USF1‐Mut had no response to the alterations of miR‐708‐5p (Figures 5B,C and S1C). RIP experiments illuminated that LOXL1‐AS1, miR‐708‐5p and USF1 were abundant in Ago2 precipitates (Figure 5D), further confirming the interaction between LOXL1‐AS1, miR‐708‐5p and USF1. Moreover, it was disclosed that the luciferase activity of USF1‐WT declined by overexpression of miR‐708‐5p was recovered by upregulation of LOXL1‐AS1; meanwhile, the promoting influences of miR‐708‐5p knockdown on the luciferase activity of USF1‐WT were abolished when LOXL1‐AS1 was silenced (Figures 5E and S1D). RT‐qPCR and Western blot elucidated that the mRNA and protein levels of USF1 were suppressed by ectopic expression of miR‐708‐5p and then renewed due to upregulation of LOXL1‐AS1 (Figure 5F). Similarly, depletion of miR‐708‐5p reinforced the mRNA and protein expression of USF1 and the rebound of USF1 levels occurred with LOXL1‐AS1 inhibition (Figure 5G). All these findings revealed that LOXL1‐AS1 acted as a ceRNA to modulate USF1 through competing for miR‐708‐5p.

### 3.6 USF1 activated SOX2 expression at the transcriptional level

**Figure 6:**
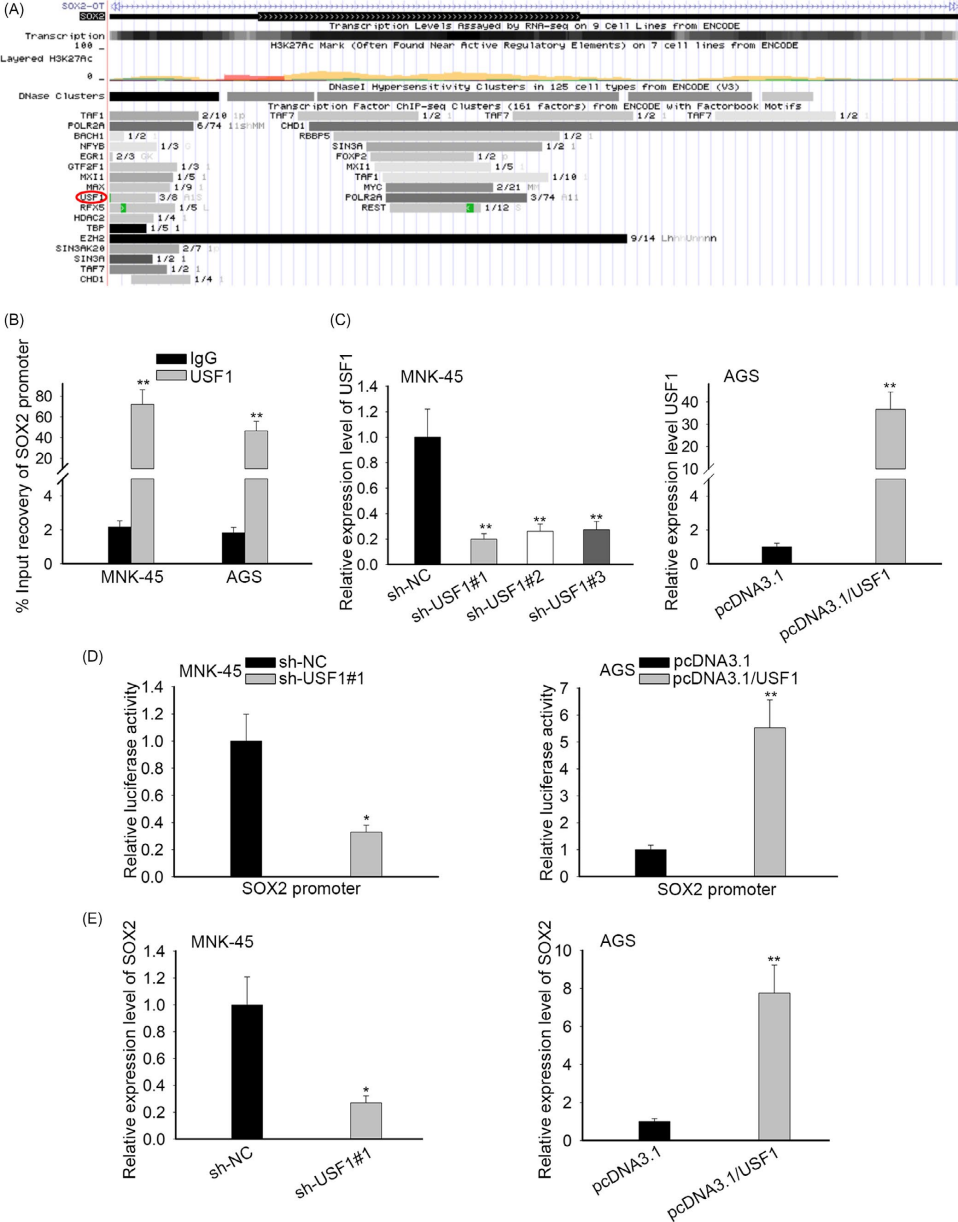
USF1 activated SOX2 expression at the transcriptional level. A, With the aid of UCSC database, it was found that USF1 might bind with the promoter region of SOX2. B, The binding capability of USF1 to SOX2 promoter was analysed by ChIP assay. C, RT‐qPCR was adopted to evaluate the transfection efficiency. D, The luciferase activity of SOX2 promoter was determined by luciferase reporter assays when USF1 was silenced or overexpressed. E, The regulation of USF1 on SOX2 expression was estimated by RT‐qPCR. ^*^ *P* < .05, ^**^ *P* < .01, ^***^ *P* < .001

Through employment of UCSC database, we found that USF1 was a transcription factor that might bind with the promoter region of SOX2 (Figure 6A). ChIP assay was carried out and unveiled that SOX2 promoter was highly expressed in compounds precipitated by USF1 compared with IgG precipitates (Figure 6B), suggesting that USF1 directly bound to SOX2 promoter. Subsequently, USF1 was knocked down in MKN‐45 cells and upregulated in AGS cells and the efficiency of transfection was verified by RT‐qPCR analysis (Figure 6C). Our data certified that suppression of USF1 resulted in the lessened luciferase activity of SOX2 and overexpression of USF1 led to the opposite consequence (Figure 6D). In concert with mentioned findings, it was proofed that the expression of SOX2 was restrained by USF1 downregulation whereas prompted by forced expression of USF1 (Figure 6E). On the whole, USF1 worked as a transcriptional activator of SOX2.

### 3.7 LOXL1‐AS1/miR‐708‐5p/USF1 pathway induced gastric cancer tumorigenesis and development

**Figure 7:**
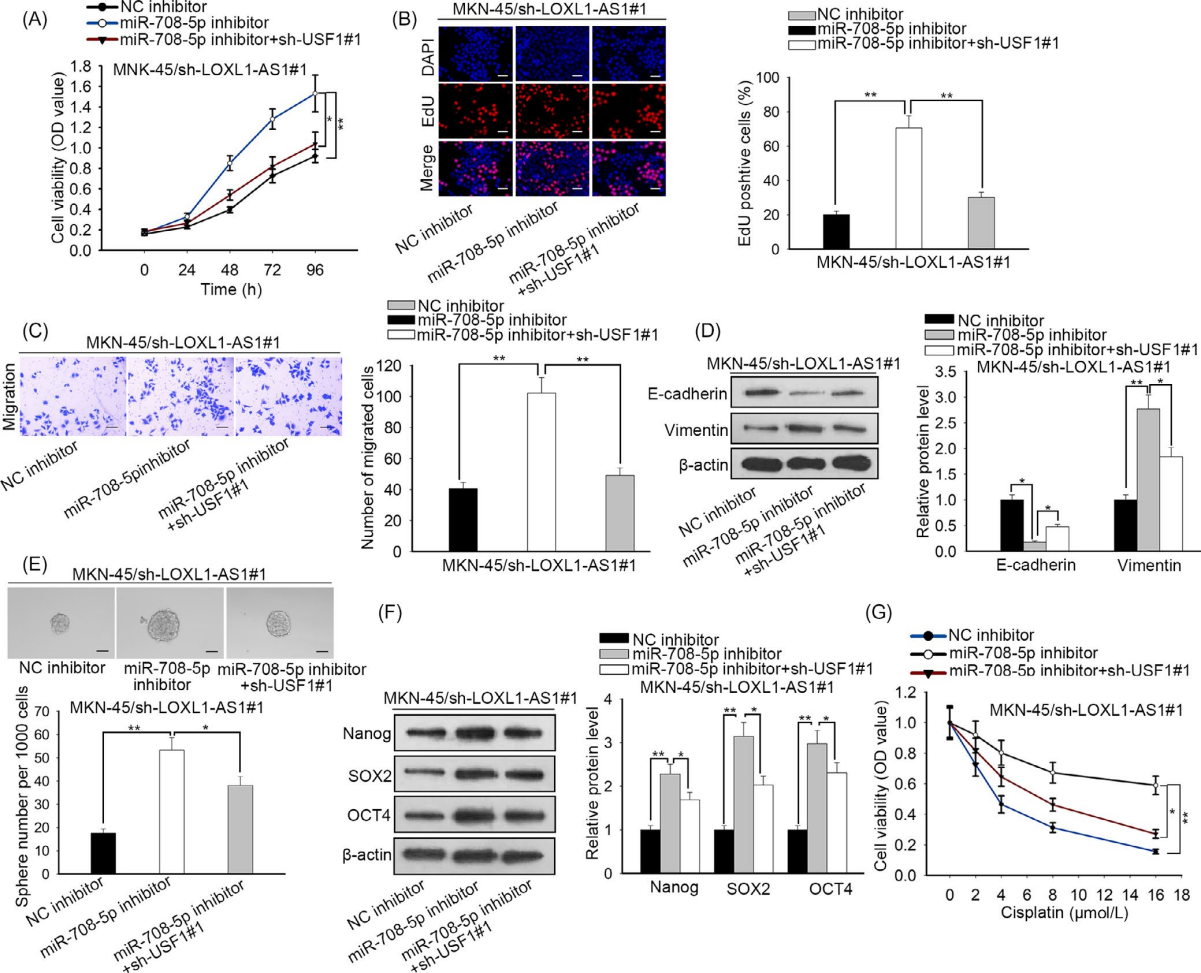
LOXL1‐AS1/miR‐708‐5p/USF1 pathway induced gastric cancer tumorigenesis and development. A, The CCK‐8 assay was employed to examine cell viability after transfection. B, The function of LOXL1‐AS1/miR‐708‐5p/USF1 in cell proliferation was further validated by EdU assay. C, The transwell assay was carried out to evaluate cell migration. D, Western blot was used to detect the expression of EMT‐related proteins E‐cadherin and vimentin. E‐F, Cell stemness was assessed by sphere formation assay and Western blot analysis. G, After transfection, cell viability of MNK‐45 cells was tested by CCK‐8 assay in the presence of different doses of cisplatin. ^*^ *P* < .05, ^**^ *P* < .01, ^***^ *P* < .001

To justify the role of LOXL1‐AS1/miR‐708‐5p/USF1 in the progression of gastric carcinoma, rescue assays were conducted. After transfection, miR‐708‐5p was silenced and USF1 was overexpressed in LOXL1‐AS1‐downregulated MKN‐45 cells (Figure S1E). CCK‐8 and EdU assays manifested that the inhibition of cell proliferation caused by miR‐708‐5p knockdown was abrogated owing to silencing of USF1 (Figure 7A,B). Transwell assay testified that cell migratory capacity was promoted by miR‐708‐5p inhibitor and retrieved by depletion of USF1 (Figure 7C). As anticipated, Western blot exposed that knockdown of miR‐708‐5p resulted in the reduced E‐cadherin expression and the elevated level of vimentin; simultaneously, the impacts of miR‐708‐5p inhibitor were counteracted by silencing of USF1 (Figure 7D). Furthermore, we observed that the heightened sphere‐forming ability induced by downregulation of miR‐708‐5p was repressed by USF1 depletion (Figure 7E). In agreement with the foregoing, the expression of Nanog, SOX2 and OCT4 increased by miR‐708‐5p inhibitor was diminished on account of USF1 knockdown (Figure 7F). Besides, the sensitization of transfected MKN‐45 cells to cisplatin was suppressed by miR‐708‐5p inhibitor and subsequently recovered by depletion of USF1 (Figure 7G). Namely, described results affirmed that LOXL1‐AS1 maintained stemness and accelerated gastric cancer deterioration via miR‐708‐5p/USF1.

### 3.8 LOXL1‐AS1 accelerated cell growth of gastric cancer in vivo

**Figure 8:**
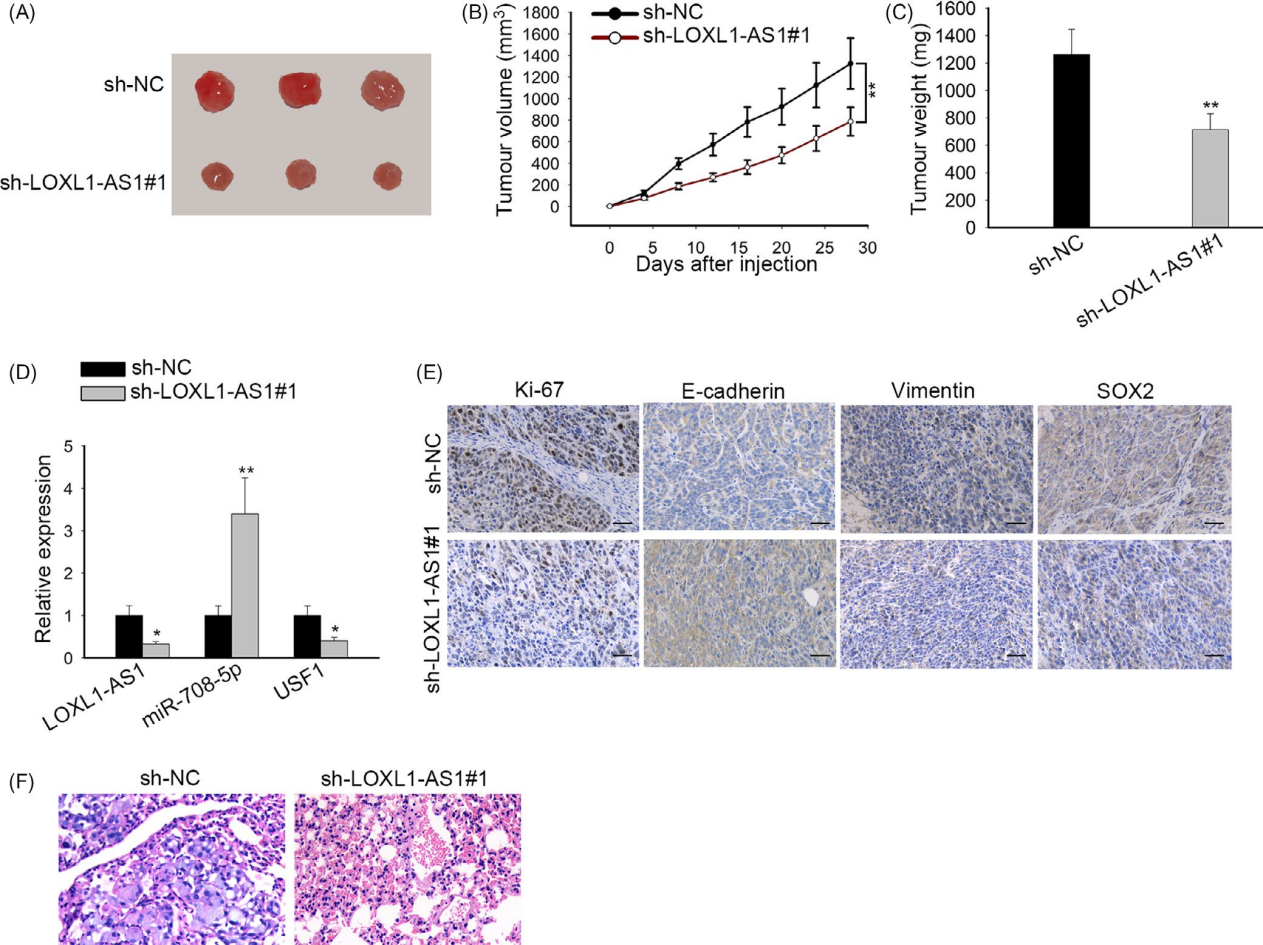
LOXL1‐AS1 accelerated cell growth of gastric cancer in vivo. A, Images of xenografts from nude mice injected with MNK‐45 cells stably transfected with sh‐NC or sh‐LOXL1‐AS1#1. B, The growth curve of neoplasms was plotted by monitoring tumour volume. C, The tumour tissues from mice were weighted at 4 wk after inoculation. D, The expression of LOXL1‐AS1, miR‐708‐5p and USF1 in xenografts was examined by RT‐qPCR. E, IHC assay was performed to assess the levels of Ki‐67, E‐cadherin, vimentin and SOX2 in neoplasms. F, Lung metastasis was measured in sh‐NC group or sh‐LOXL1‐AS1#1 group. ^ *^ *P* < .05, ^***^ *P* < .001

To further validate the carcinogenic function of LOXL1‐AS1 in vivo, we implemented animal experiments. Nude mice were randomly divided into two groups, sh‐NC group and sh‐LOXL1‐AS1#1 group. Then, designated transfected cells were subcutaneously injected into corresponding mice. It was indicated that the size and weight of xenografts formed by MKN‐45 sh‐LOXL1‐AS1#1 cells were much smaller than those formed by MKN‐45 sh‐NC cells (Figure 8A‐C). In addition, RT‐qPCR analysis demonstrated that knockdown of LOXL1‐AS1 contributed to the decrease of LOXL1‐AS1 and USF1 expression and the enhancement of miR‐708‐5p level in neoplasms from mice (Figure 8D). IHC assay illustrated that silencing of LOXL1‐AS1 prominently inhibited the expression of Ki‐67, vimentin and SOX2 while fortified E‐cadherin level (Figure 8E), further unveiling the stimulative role of LOXL1‐AS1 in cell proliferation, EMT and stemness in vivo. In short, we proved that LOXL1‐AS1 promoted gastric cancer development in vivo.

## 4 DISCUSSION

Gastric cancer is characterized as one of the most frequent malignant tumours in human digestive systems, with the fourth highest morbidity and the third highest fatality rate among all malignancies worldwide.^23^, ^24^ The delayed diagnosis of gastric cancer often occurs in the great mass of patients attributable to the lack of definite clinical symptoms and sensitive biomarkers at early stage.^25^, ^26^ In spite of great advance in the clinical therapy, the 5‐year survival rate of patients with advanced gastric cancer remains unfavourable.^27^ As a result, the deeper understanding of the pathological mechanism underlying gastric carcinoma is imperative for the development of treatments for this disease.

A myriad of literatures have expounded that lncRNAs serve as important regulators in a wide range of malignancies by modulating various cell processes, such as cell proliferation, metastasis, drug resistance and stemness.^28^, ^29^ For instance, lncRNA MALAT1 promotes gastric cancer cell stemness through fortifying SOX2 mRNA stability.^30^ LncRNA LINC01197 regulated by FOXO1 inhibits Wnt/β‐catenin signalling to retard cell proliferation of pancreatic adenocarcinoma.^31^ LncRNA KCNQ1OT1 restrains the sensitivity of colon cancer cells to oxaliplatin via regulation of miR‐34a/ATG4B pathway.^32^ The tumour‐promoting role of LOXL1‐AS1 has been confirmed in multiple cancers, including glioblastoma,^33^ cholangiocarcinoma,^34^ prostate cancer 16 and osteosarcoma.^15^ Nevertheless, the biological function of LOXL1‐AS1 in gastric cancer is still an emerging field to be explored. In the present study, we found that LOXL1‐AS1 expression was overtly upregulated in gastric cancer tissues and cells. In addition, high expression of LOXL1‐AS1 was closely correlated with poor prognosis of gastric cancer. Functional experiments demonstrated that LOXL1‐AS1 promoted cell proliferation, migration, EMT and the maintenance of CSC characteristics in gastric carcinoma.

Upstream stimulating factor 1 (USF1) is a critical component of the basic helix‐loop‐helix leucine zipper (bHLH‐LZ) family of transcription factors.^35^ The HLH‐LZ structure contained in USF1 can combine with the E‐box of many gene promoter regions.^36^ Therefore, USF1 involves in the transcription process of numerous proteins and works as an important regulatory factor in plenty of diseases, including cancer.^37^, ^38^ In contrast with normal tissues and cells, the remarkable upregulation of USF1 expression was observed in gastric cancer samples and cells. Besides, it was suggested that the mRNA and protein levels of USF1 were negatively regulated by LOXL1‐AS1. Mechanism researches exposed that LOXL1‐AS1 protected USF1 from miR‐708‐5p‐mediated degradation and USF1 contributed to the transcriptional activation of SOX2. Further assays testified that LOXL1‐AS1 maintained CSC properties and induced gastric cancer tumorigenesis by targeting miR‐708‐5p/USF1 pathway. The carcinogenic role of LOXL1‐AS1 in vivo was also verified by animal experiments.

To summarize, this study was the first to elucidate the function and regulatory mechanism of LOXL1‐AS1 in gastric carcinoma. Our results illustrated that LOXL1‐AS1 regulated USF1 to execute its oncogenic activities in gastric cancer through sponging miR‐708‐5p, which opened a novel prospective for the therapeutic regimens of patients with gastric cancer. In the future, in‐depth studies are to be unfolded to probe whether SOX2 mediates the role of LOXL1‐AS1/miR‐708‐5p/USF1 pathway.

## CONFLICTS OF INTEREST

None.

## Data Availability

Research data are not shared.
