# Supplementary figures and images for: LncRNA LOXL1‐AS1 facilitates the tumorigenesis and stemness of gastric carcinoma via regulation of miR‐708‐5p/USF1 pathway
Source: Cell Prolif. 2019 Aug 29;52(6):e12687. doi: 10.1111/cpr.12687 (PMC6869681; doi:10.1111/cpr.12687)

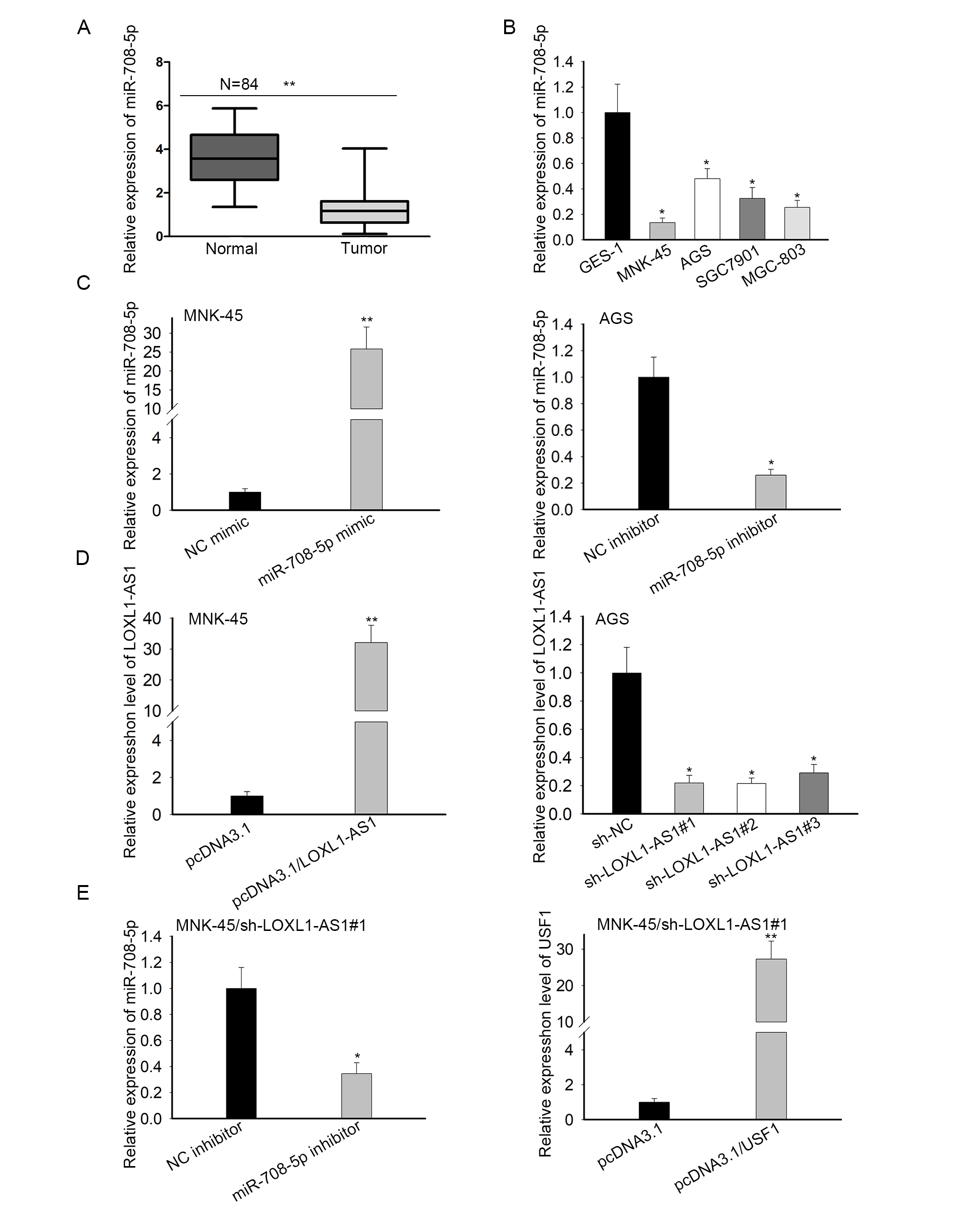

Supplement: Supplementary file 1 [file CPR-52-e12687-s001.tif]
